# Supplementary material for: The Application of ATR-FTIR Spectroscopy and the Reversible DNA Conformation as a Sensor to Test the Effectiveness of Platinum(II) Anticancer Drugs
Source: Sensors (Basel). 2018 Dec 6;18(12):4297. doi: 10.3390/s18124297 (PMC6308638; doi:10.3390/s18124297)
Supplement: Supplementary file 1 [file sensors-18-04297-s001.pdf]

Supplementary Material to:

The Application of ATR-FTIR Spectroscopy and the Reversible DNA Conformation as a Sensor to Test the Effectiveness of Platinum(II) Anticancer Drugs

Khansa Al-Jorani , Anja Rüther , Miguela Martin , Rukshani Haputhanthri , Glen B. Deacon , Hsiu Lin Li and Bayden R. Wood*

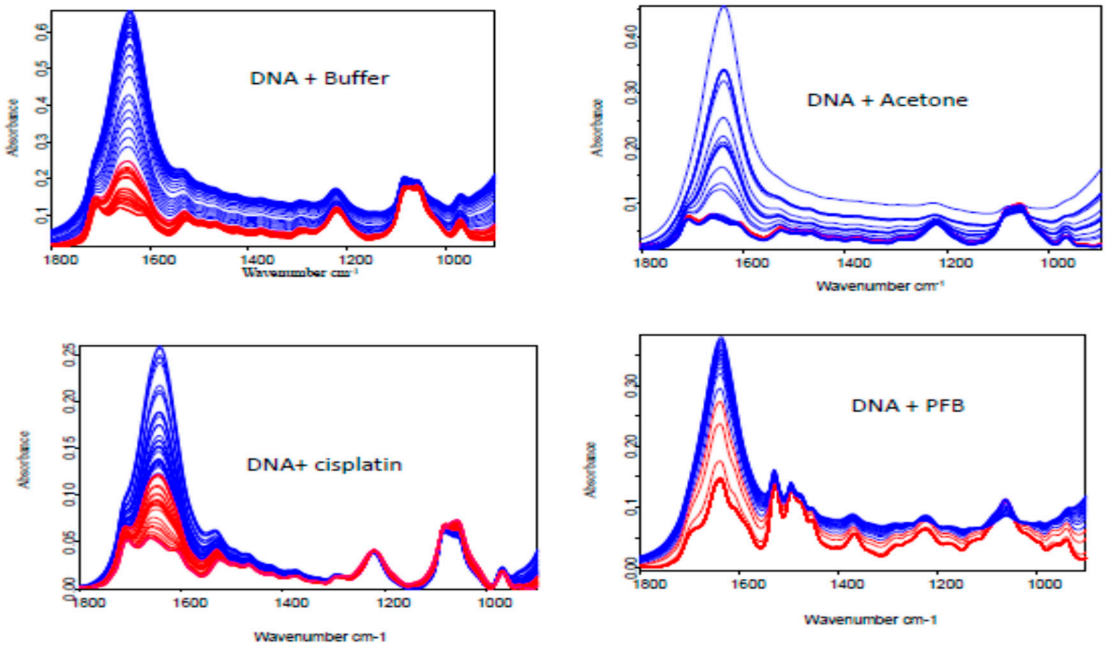

Figure S1. FTIR-IR absorbance spectra of the control with buffer and acetone, DNA-drug samples with cisplatin and PFB.

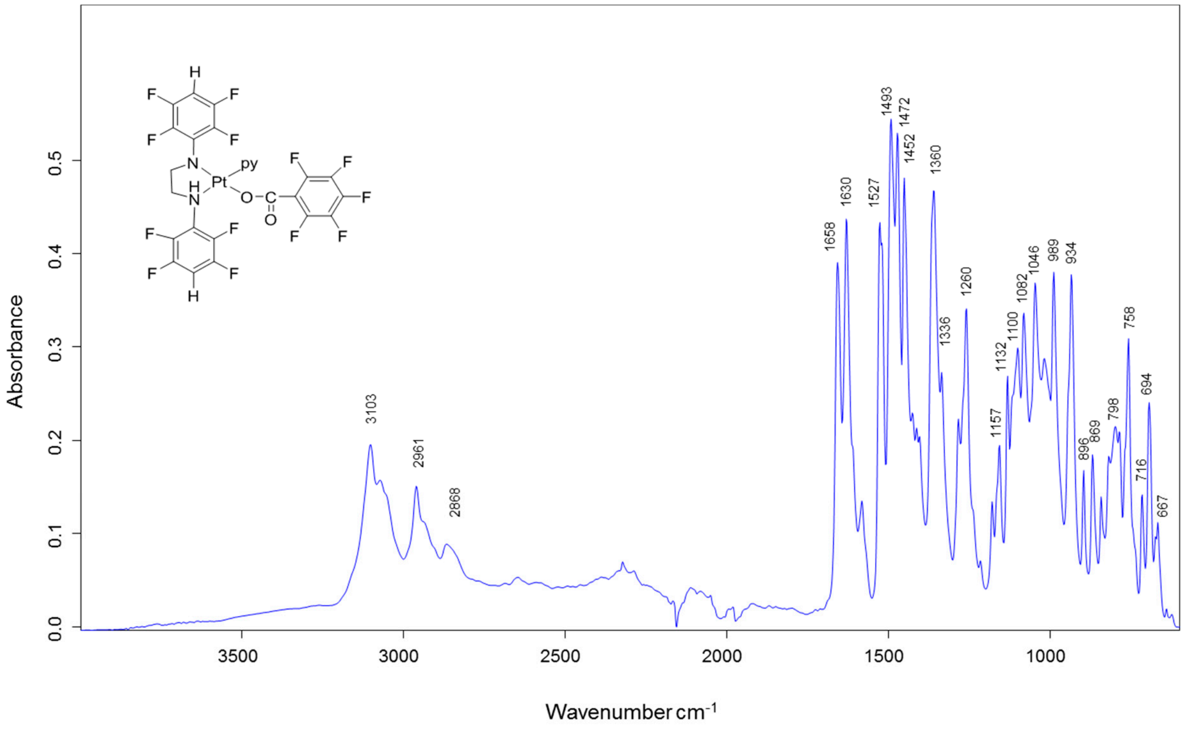

Figure S2. ATR-IR spectrum of PFB.

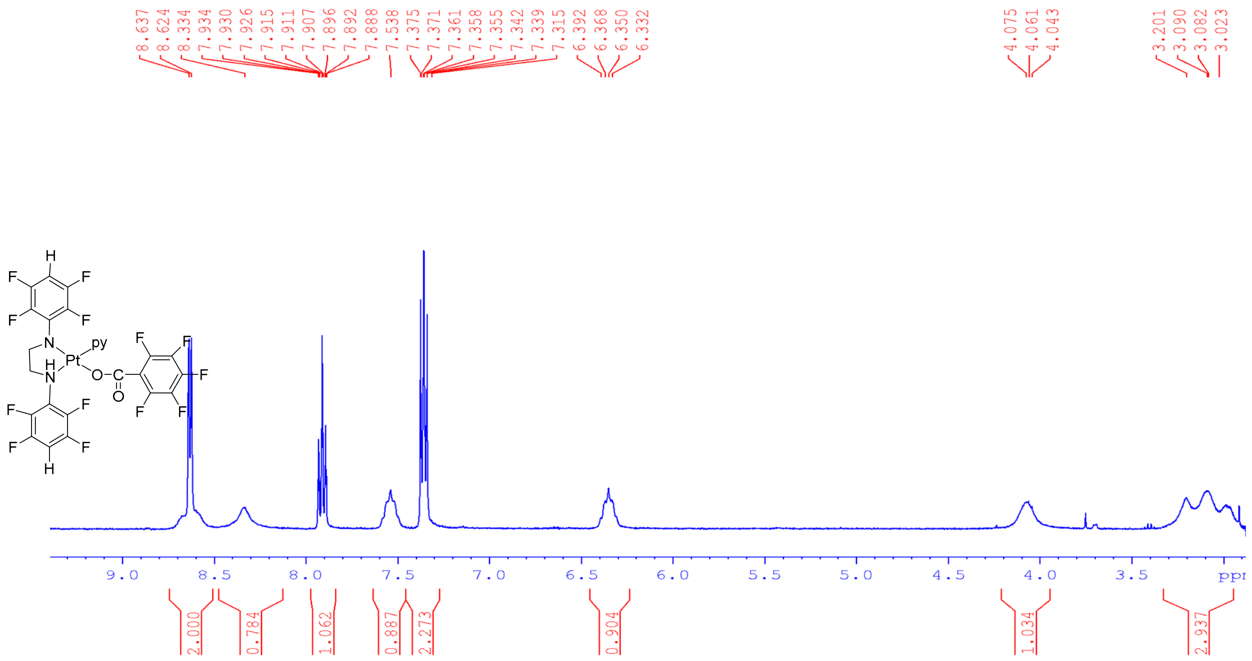

Figure S3. ^{1}HNMR spectrum of PFB.

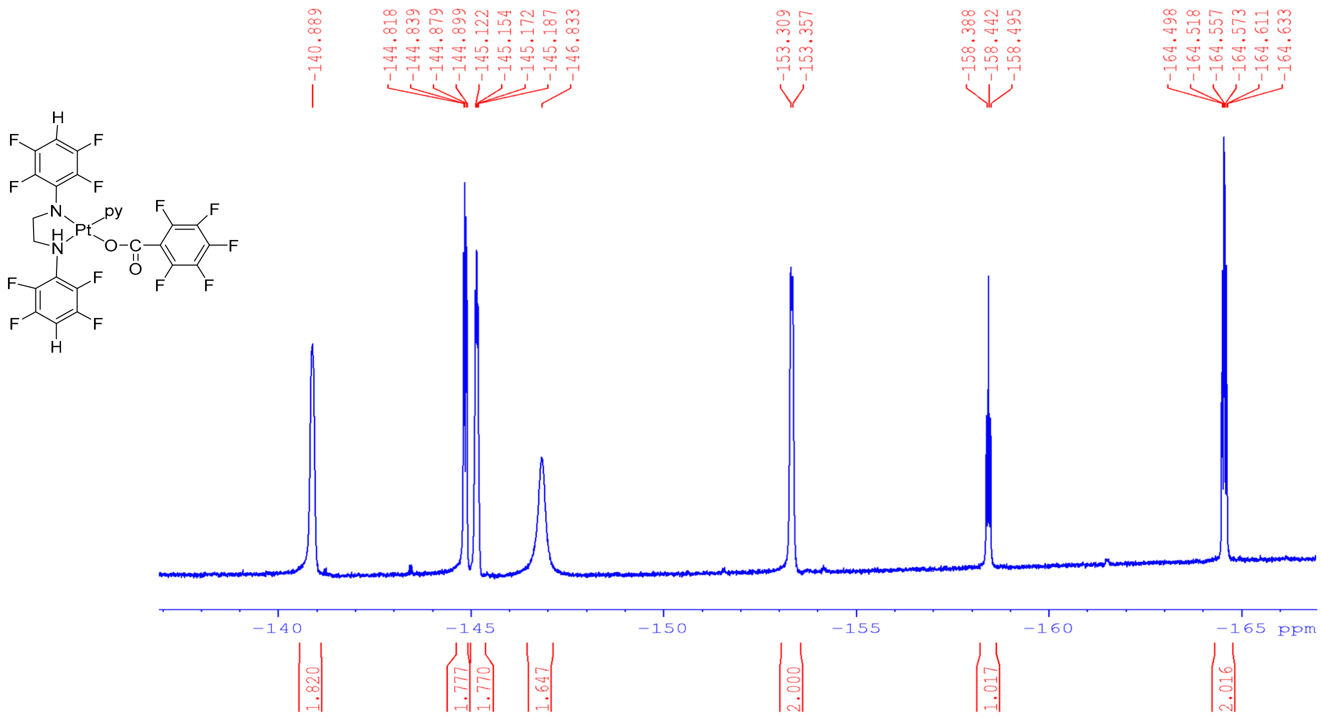

Figure S4. ^{19}FNMR spectrum of PFB.

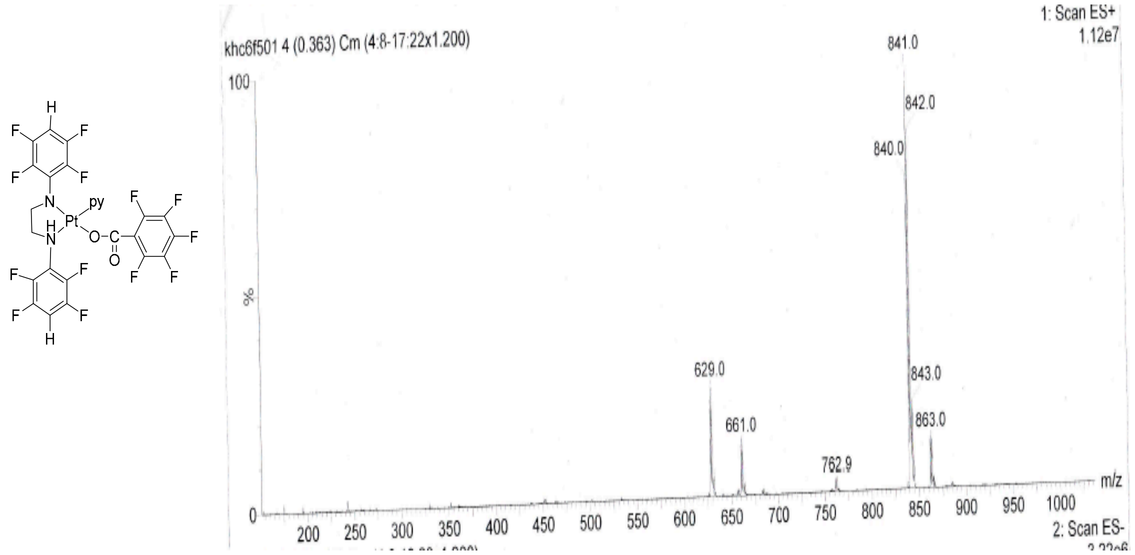

Figure S5. $ESMS^+$ spectrum of PFB.

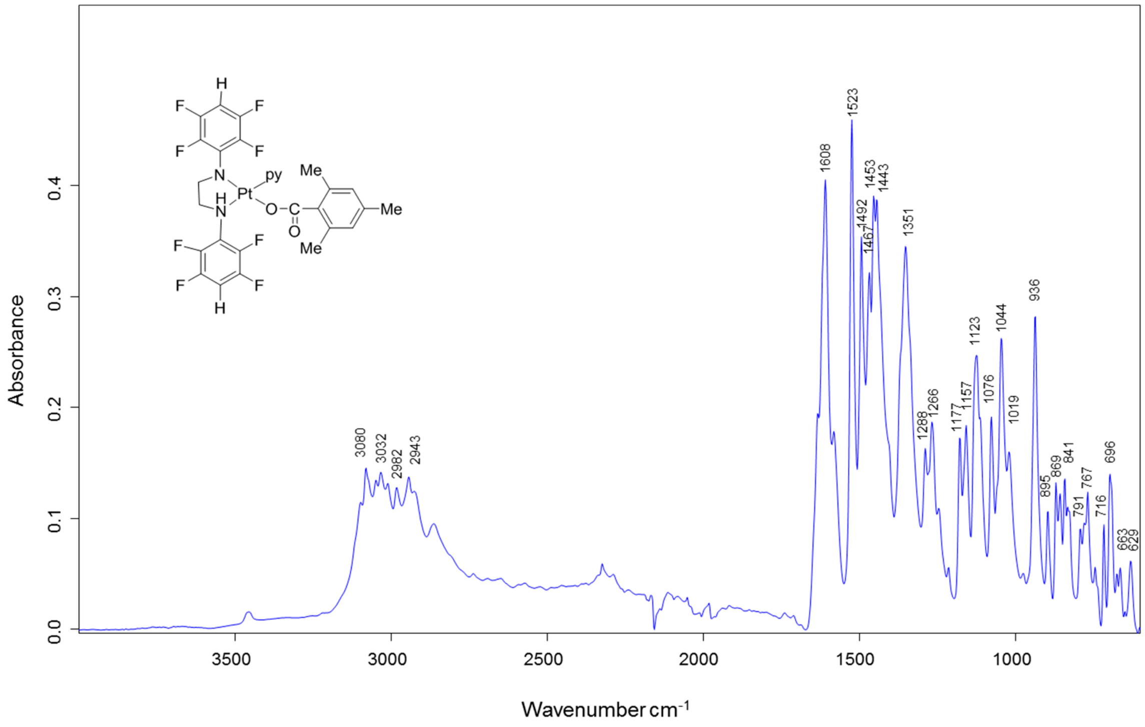

Figure S6. ATR-IR spectrum of TMB.

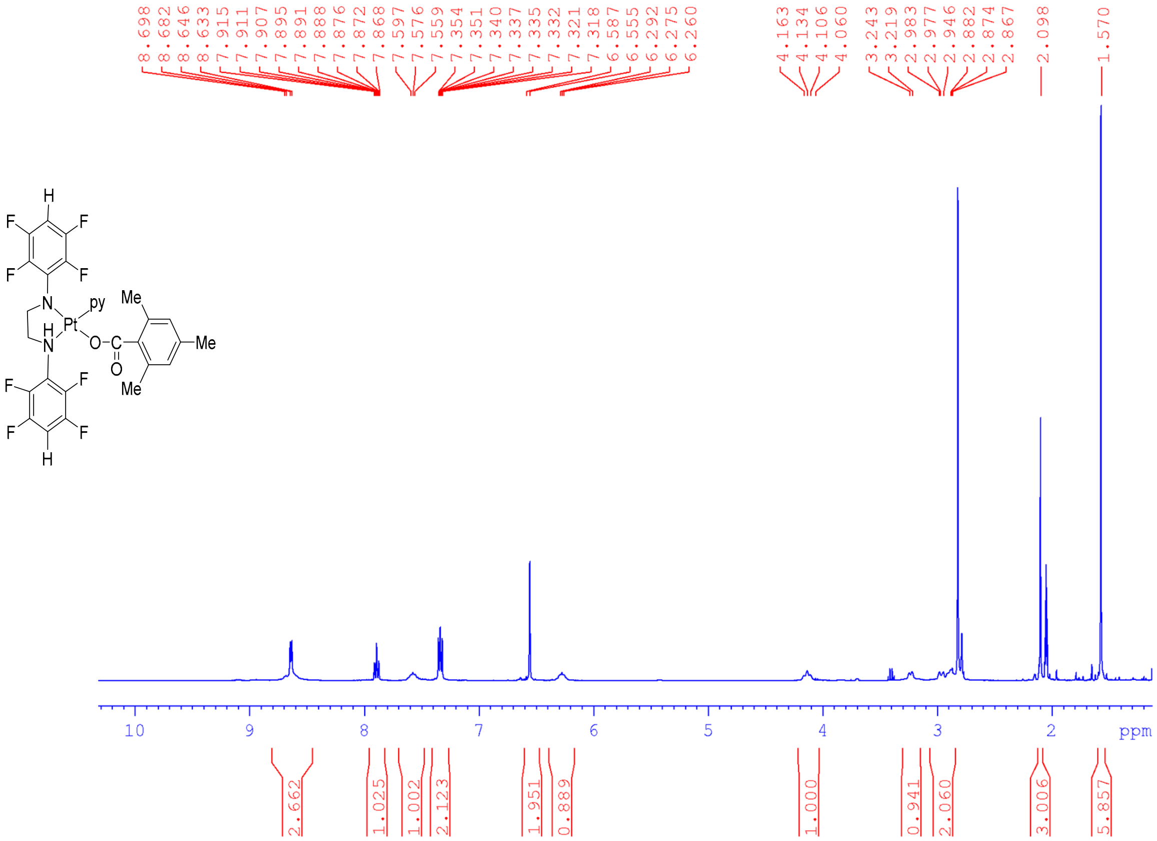

Figure S7. ^{1}HNMR spectrum of TMB.

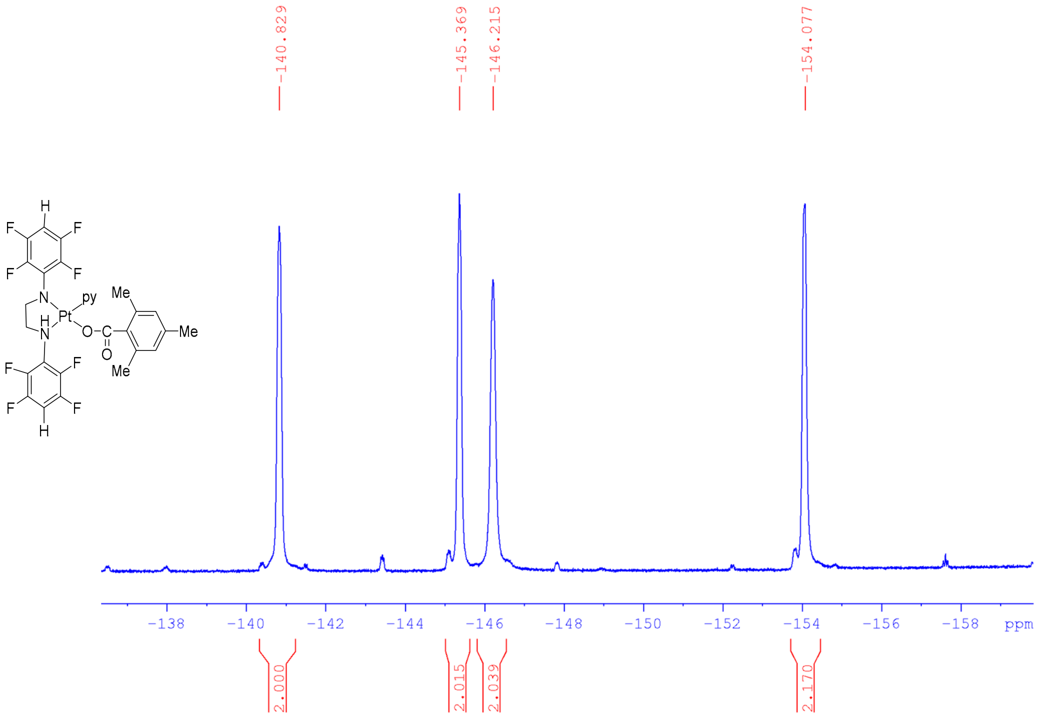

Figure S8. ^{19}FNMR spectrum of TMB.

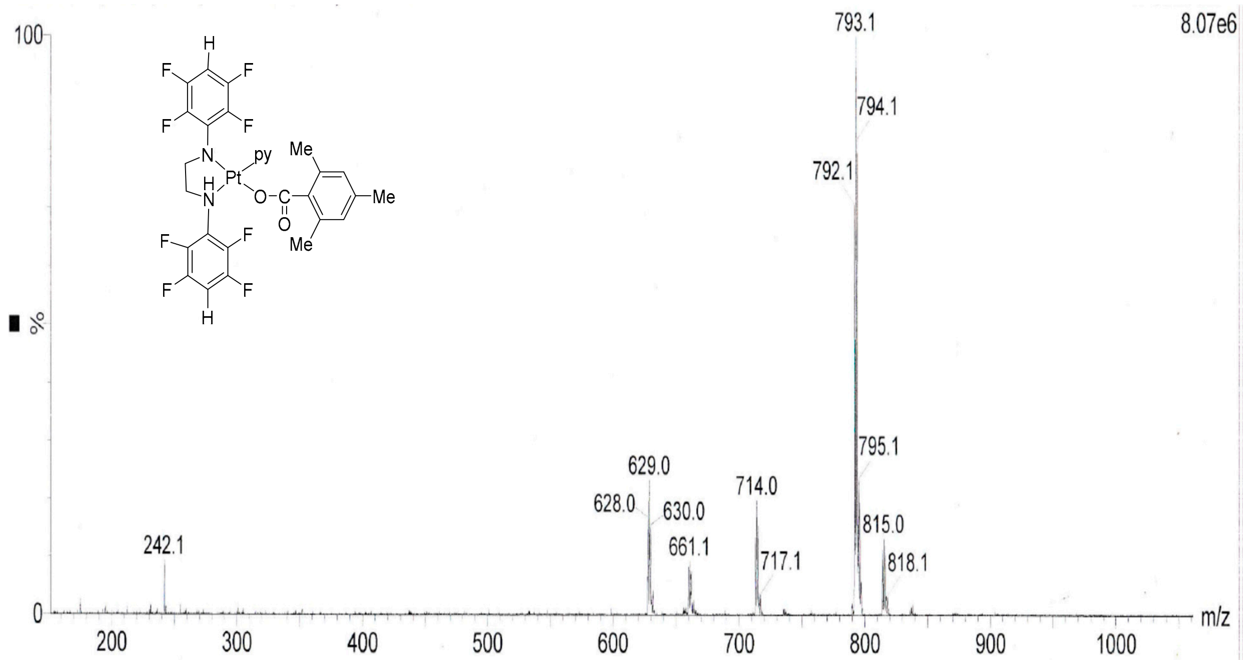

Figure S9. ESMS$^+$ spectrum of TMB.
